# Multi-Omics Characterization of Tumor Microenvironment Heterogeneity and Immunotherapy Resistance Through Cell States–Based Subtyping in Bladder Cancer

**DOI:** 10.3389/fcell.2021.809588

**Published:** 2022-02-09

**Authors:** Rixin Hu, Tao Tao, Lu Yu, Qiuxia Ding, Guanghui Zhu, Guoyu Peng, Shiwen Zheng, Leyun Yang, Song Wu

**Affiliations:** ^1^ Health Science Center, School of Basic Medical Sciences, Shenzhen University, Shenzhen, China; ^2^ Department of Urology, The Affiliated Luohu Hospital of Shenzhen University, Shenzhen University, Shenzhen, China; ^3^ Shenzhen Following Precision Medical Research Institute, Luohu Hospital Group, Shenzhen, China; ^4^ Teaching Center of Shenzhen Luohu Hospital, Shantou University Medical College, Shantou, China; ^5^ Department of Urology, South China Hospital, Health Science Center, Shenzhen University, Shenzhen, China

**Keywords:** multi-omics, bladder cancer, tumor microenvironment, cell states, subtype, heterogeneity, immunotherapy resistance, prognosis

## Abstract

Due to the strong heterogeneity of bladder cancer (BC), there is often substantial variation in the prognosis and efficiency of immunotherapy among BC patients. For the precision treatment and assessment of prognosis, the subtyping of BC plays a critical role. Despite various subtyping methods proposed previously, most of them are based on a limited number of molecules, and none of them is developed on the basis of cell states. In this study, we construct a single-cell atlas by integrating single cell RNA-seq, RNA microarray, and bulk RNA-seq data to identify the absolute proportion of 22 different cell states in BC, including immune and nonimmune cell states derived from tumor tissues. To explore the heterogeneity of BC, BC was identified into four different subtypes in multiple cohorts using an improved consensus clustering algorithm based on cell states. Among the four subtypes, C1 had median prognosis and best overall response rate (ORR), which characterized an immunosuppressive tumor microenvironment. C2 was enriched in epithelial-mesenchymal transition/invasion, angiogenesis, immunosuppression, and immune exhaustion. Surely, C2 performed the worst in prognosis and ORR. C3 with worse ORR than C2 was enriched in angiogenesis and almost nonimmune exhaustion. Displaying an immune effective environment, C4 performed the best in prognosis and ORR. We found that patients with just an immunosuppressive environment are suitable for immunotherapy, but patients with an immunosuppressive environment accompanied by immune exhaustion or angiogenesis may resist immunotherapy. Furthermore, we conducted exploration into the heterogeneity of the transcriptome, mutational profiles, and somatic copy-number alterations in four subtypes, which could explain the significant differences related to cell states in prognosis and ORR. We also found that PD-1 in immune and tumor cells could both influence ORR in BC. The level of TGFβ in a cell state can be opposite to the overall level in the tissues, and the level in a specific cell state could predict ORR more accurately. Thus, our work furthers the understanding of heterogeneity and immunotherapy resistance in BC, which is expected to assist clinical practice and serve as a supplement to the current subtyping method from a novel perspective of cell states.

## Introduction

Around the world, there were a total of 5,73,278 new cases and 2,12,536 new deaths linked to bladder cancer (BC) in 2020 (Sung et al., 2021). In the United States alone, there were 83,730 new cases and 17,200 new deaths from BC reported, which is predominant in new cases and new deaths of urologic tumors (Siegel et al., 2021). Previously, the treatments and drugs available for BC were quite limited. Some patients even showed intolerance to the toxicity of platinum-based chemotherapy, thus rendering the prognosis of BC relatively poor.

However, the emergence of immunotherapy has made immune checkpoint blockades applicable to BC patients, especially those with intolerance to platinum-based chemotherapy. Currently, there have been five immune checkpoint inhibitors approved for metastatic BC and adopted as the standard second-line treatment for BC after the failure of platinum-based chemotherapy. In spite of this, the proportion of BC patients responding to immunotherapy reaches as low as about 20%, which is probably attributed to the heterogeneity of the tumor microenvironment (TME). However, discovering the relationship between the heterogeneity of TME and immunotherapy resistance against BC and identifying those patients fit for immunotherapy remain arduous tasks. To solve this problem and obtain a deeper understanding of heterogeneity in BC, there are various subtyping methods proposed. In histology, BC can be subtyped into NMIBC and MIBC, depending on the pathological features and molecular characteristics. Based on DNA, somatic copy-number alterations (SCNAs), and methylation profiles, subtypes are classified and some of them associated with prognosis (Hurst et al., 2012; Aine et al., 2015). TCGA-BLCA defined five subtypes, including luminal, luminal_infiltrated, luminal_papillary, basal_squamous, and neuronal; the basal subtype is usually associated with a bad prognosis, whereas luminal_papillary is associated with a good prognosis (Robertson et al., 2017).

Despite these previously proposed subtyping methods, most of them are based on a limited number of molecules, which leads to the lack of a subtyping method based on cell states. As a part of TME, peritumor cells are closely related to tumor initiation, progression, recurrence, and drug response, and cell states are more comprehensive than molecules. Since then, the subtypes based on cell states may be more effective in revealing the relationship between the heterogeneity of TME and immunotherapy resistance, identifying therapeutic targets, and assisting in the selection of treatment and assessment of prognosis in BC. To be specific, single cell RNA-seq makes it possible to provide detailed information about cell states. Therefore, BC can be divided into subtypes with distinct heterogeneity based on cell states through the combination of single cell RNA-seq, RNA microarray, and bulk RNA-Seq.

In our work, a single-cell atlas consisted of 22 immune and nonimmune cell states in BC was constructed by means of reanalyzing single-cell RNA-seq data. Then, the CIBERSORTx algorithm was adopted to identify the cell states of BC in multiple cohorts, and BC was divided into four subtypes according to the levels of 22 cell states. To verify that our subtypes classified by cell states can be relied on to distinguish the heterogeneity of BC and guide the treatment, the molecular, genomic, and clinical characteristics of the four subtypes were evaluated thoroughly. Besides this, our cell-states subtyping method was compared with other subtyping methods. According to these results, the cell-states subtyping method proposed in this study can effectively identify heterogeneity in BC TME and discover the relationship between the heterogeneity of TME and immunotherapy resistance. Our work is expected to assist in the selection of appropriate therapy for BC patients and contribute a supplement to the existing subtyping methods.

## Materials and Methods

### Cohort Collection

A total of seven BC cohorts were retrieved from the GEO and SRA databases, including tumor samples from the TCGA BC project (TCGA-BLCA). Among these cohorts, a single cell RNA-seq cohort was used to obtain cell states in BC, three cohorts (UROMOL, GSE13507, and GSE48276) were utilized for BC cell-state subtype construction, and three cohorts (TCGA-BLCA, GSE31684, and IMvigor210) were used for BC classification with the cell-state subtyping method. Besides this, immunotherapy response among subtypes was explored in an immunotherapy cohort (IMvigor210). The single-cell RNA-seq data of BC was downloaded from SRA^1^ under the accession code PRJNA662018. The gene expression profiling, somatic mutation, SCNAs, and clinical data of TCGA-BLCA tumor tissues were downloaded from the GDC portal^2^. Moreover, gene expression profiles and clinical data of tumor tissues in GSE13507, GSE48276, and GSE31684 cohorts were downloaded from GEO^3^. Gene expression profiles and clinical data of the UROMOL cohort were downloaded from ArrayExpress^4^. Finally, gene expression profiles and clinical data of the IMvigor210 cohort were obtained from the supplement of Turley et al. (Mariathasan et al., 2018). The inclusion criteria for the samples were as follows. In the TCGA-BLCA cohort, 430 samples were obtained from GDC. Then, 19 normal samples, six blood or duplicated samples were excluded. Finally, 405 tumor samples from BC tissues were obtained to identify subtypes. In the UROMOL cohort, 406 tumor samples were all obtained from the discovery cohort, and all were used to identify subtypes. In the IMvigor210 cohort, 348 tumor samples were acquired from several organs, and 195 tumor samples from BC were included to identify subtypes. Among 195 samples, 168 tumor samples had the information of immunotherapy and were used to explore immunotherapy resistance. In GSE13507, 256 samples were downloaded from GEO; we then excluded samples from normal and tumor mucosae and finally obtained 188 samples from tumor tissue to identify subtypes. In GSE31684, we got 93 BC samples, and all were used to identify subtypes. In GSE48276, there were 116 BC samples, and all were used to identify subtypes.

### Construction of a Single-Cell Atlas in BC and Identification of Cell States in Multiple Cohorts

The raw data of eight BC samples were processed and mapped reads to the transcriptome by using Cell Ranger 6.1. After getting a raw unique molecular identifier (UMI) count matrix, SeuratV4.0 (Hao et al., 2021) was employed to quality control the eight data sets based on the following quality control criteria: UMI<200, UMI>5000, and mitochondrial gene content >20% were removed. Then, integration of eight data sets was performed by the function “IntegrateData,” and batch effects were removed using the SCTransform algorithm in Seurat. Finally, the remaining 70,387 cells were applied to SignacX 2.2.4 (Chamberlain et al., 2021), a neural network–based approach to identify cell states. Next, the cell states were further validated based on the CellMarker database (Zhang et al., 2019), followed by subclustering to obtain 22 cell states, which were visualized with UMAP plots. Markers of 22 cell states were identified by the function “FindAllMarkers” of Seurat and the top two markers of each cell state were displayed using Seurat’s “DimPlot.” Microarray and RNA-seq data were treated with normalization based on the instruction of CIBERSORTx (Newman et al., 2019), which were used to deconvolute to acquire the absolute proportion of 22 cell states in an absolute mode with 100 permutations, and the results with *p* < .05 were retained for the next analysis.

### Identification of BC Subtypes by Clustering Cell States

To identify BC subtypes, the R package CrossICC (Zhao et al., 2020) was applied to cluster all three cohorts (UROMOL, GSE13507, and GSE48276) with the following parameters: skip. mfs = F, max. iter = 100, pItem = 0.95, pFeature = 1, max. K = 8, cross = “cluster”, fdr. cutoff = 0.1, clusterAlg = "hc”, distance = "spearman”, cc. seed = 47, ebayes. cutoff = 0.1, and filter. cutoff = 0.1. Although the cohorts were from multiple platforms, the cohorts could be processed, and batch effects that arise from the various cohorts could be filtered out by using CrossICC, and the optimal number of subtypes can be determined using a built-in consensus clustering algorithm. Then, the clustering patterns of the three cohorts were utilized to classify other three cohorts (TCGA-BLCA, GSE31684, and IMvigor210) using the function “predictor” of CrossICC, and the BC subtypes were presented with R package pheatmap. Finally, the line graph was employed to exhibit the absolute proportion of cell states among the four subtypes in multiple cohorts.

### Characterization of TME, Inflammation, and Immunotherapy Heterogeneity by Signature in Four Subtypes

First, counts or FPKM data downloaded from TCGA-BLCA, UROMOL, and IMvigor210 were transformed to TPM data. Next, the function “calculate_sig_score” of IOBR (Zeng et al., 2021) was used to calculate TPM data to obtain signatures of these three cohorts, the function “ggboxplot” of the ggpbur package was performed to compare the signature among subtypes, and the function “stat_compare_means” was utilized to evaluate the significance of difference among subtypes, with *p* < .05 noted as *, *p* < .01 noted as **, and *p* < .001 noted as ***. The line graph was used to present the difference of the mean value of six checkpoints among four subtypes. Hierarchical bar charts were finally plotted to exhibit the PD-1 level in tumor cells and immune cells.

### Characterization of Mutation Profiles and SCNAs in Four Subtypes

The package maftools was used to analyze the mutation profiles of the TCGA-BLCA cohort, and the mutation rate of genes was presented by the function “oncoplot,” followed by identification of driver mutations in each subtype using the function “oncodrive” and exhibition with the function “plotOncodrive.” The Kaplan–Meier plot was utilized for analyzing some driver mutations in cBioPortal for Cancer Genomics^5^. The relationship between subtypes and clinical features was explored by plotting hierarchical bar charts in cBioPortal for Cancer Genomics. GISTIC2.0 (Mermel et al., 2011) was performed to analyze the copy number files from TCGA-BLCA and plotted recurrent SCNAs area, G-scores, average SCNAs amplitude, and average SCNAs frequency. Finally, survival difference caused by gene expression related to subtype-specific SCNAs was displayed by the Kaplan–Meier plot in GEPIA2^6^.

### Statistical Analysis

The analysis of our study was done under R software version 3.6.3 or 4.03 according to the requirements of the R package. The ANOVA and Kruskal–Wallis tests were used to compare normally and nonnormally distributed data for each of the four subtypes. Contingency tables were analyzed by using the Fisher’s exact or Chi-square test, and FDR was assessed by using the Benjamini–Hochberg method for multiple hypothesis testing.

## Results

### Construction of a Single-Cell Atlas in BC and Identification of Cell States in Multiple Cohorts

**FIGURE 1:**
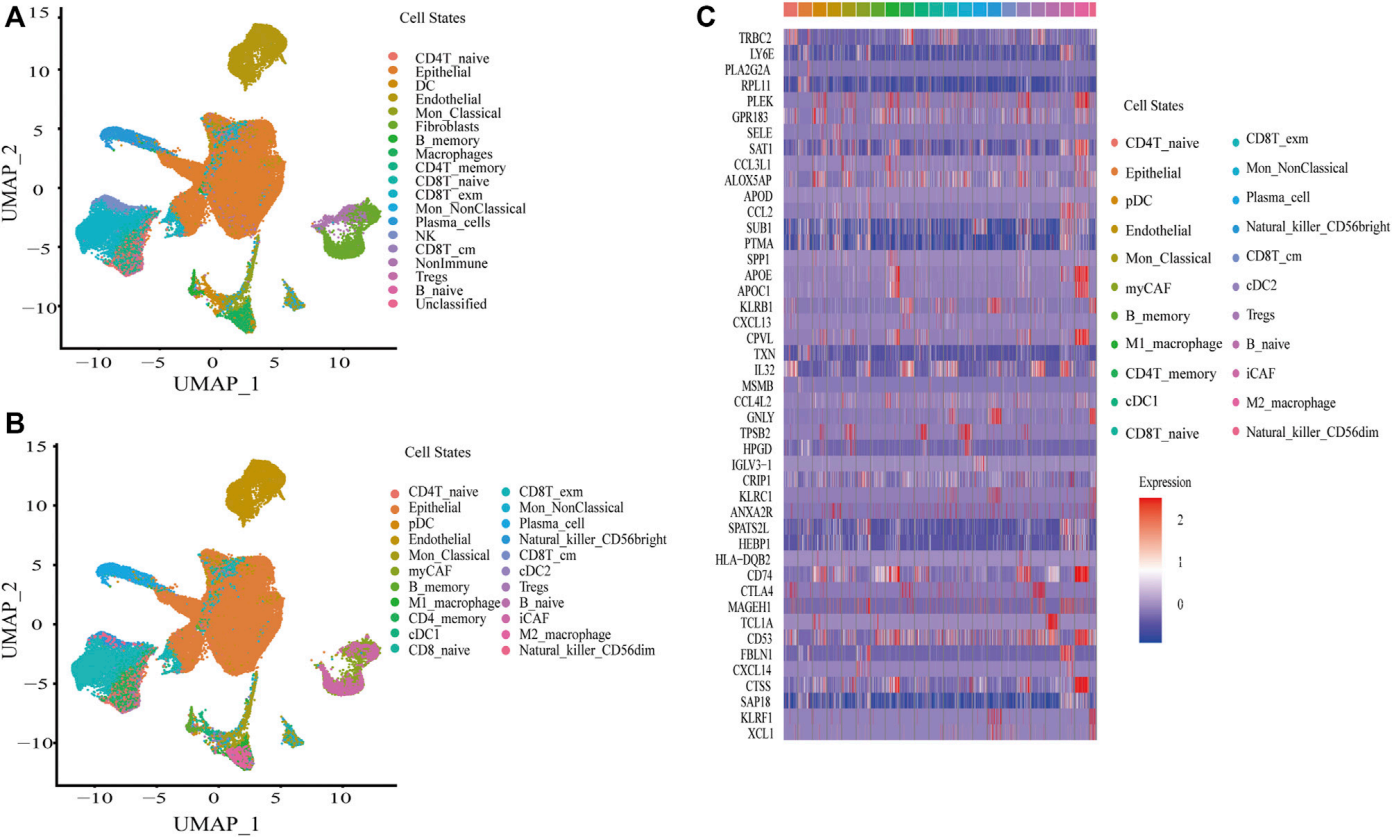
Construction of a single-cell atlas in BC. **(A)** UAMP plot of a single cell atlas of BC in first identification by Signacx. **(B)** UAMP plot of a single cell atlas of BC in subclustering. **(C)** Heatmap plot of the top two markers of cell states in BC.

The single cell RNA-seq data of eight BC samples were downloaded and reanalyzed with CellRanger, followed by quality control and removal of batch effects of the single-cell RNA-seq data with Seurat as described in Materials and Methods. After that, a total of 70,387 cells were included in this study. Signacx, a framework using neural networks, which was said to be good at identifying cell states, was used to initially identify the cells into 16 cell states, including immune cells (dendritic cell (DC), classical monocyte (Mon_Classical), non-classical monocyte (Mon_NonClassical), macrophage, CD4T_ naive, CD4T_memory, CD8T_naive, exhausted memory CD8T (CD8T_exm), central memory CD8T (CD8T_cm), regulatory T cells (Tregs), B_naive, B_memory, plasma_cell, natural killer cells (NK)), and nonimmune cells (endothelial and fibroblasts), and some other nonimmune cells had not been yet identified (Figure 1A). Subsequently, the macrophage was subclustered into M1_macrophage and M2_macrophage; DC was subclustered into pDC, cDC1, and cDC2; NK was subclustered into Natural_killer_CD56bright and Natural_killer_CD56dim; and fibroblasts were subclustered into myCAF and iCAF based on the Cellmarker database (Figure 1B). The markers of each cell state were identified by Seurat (Supplementary Table S1), and the top two markers of each cell state were displayed by heatmap (Figure 1C). Finally, TCGA-BLCA, UROMOL, GSE13507, GSE48276, GSE31684, and IMvigor210 cohorts were deconvoluted to obtain the absolute proportion of 22 cell states by using CIBERSORTx (Supplementary Table S2).

### Identification of BC Subtypes by Clustering Cell States

**FIGURE 2:**
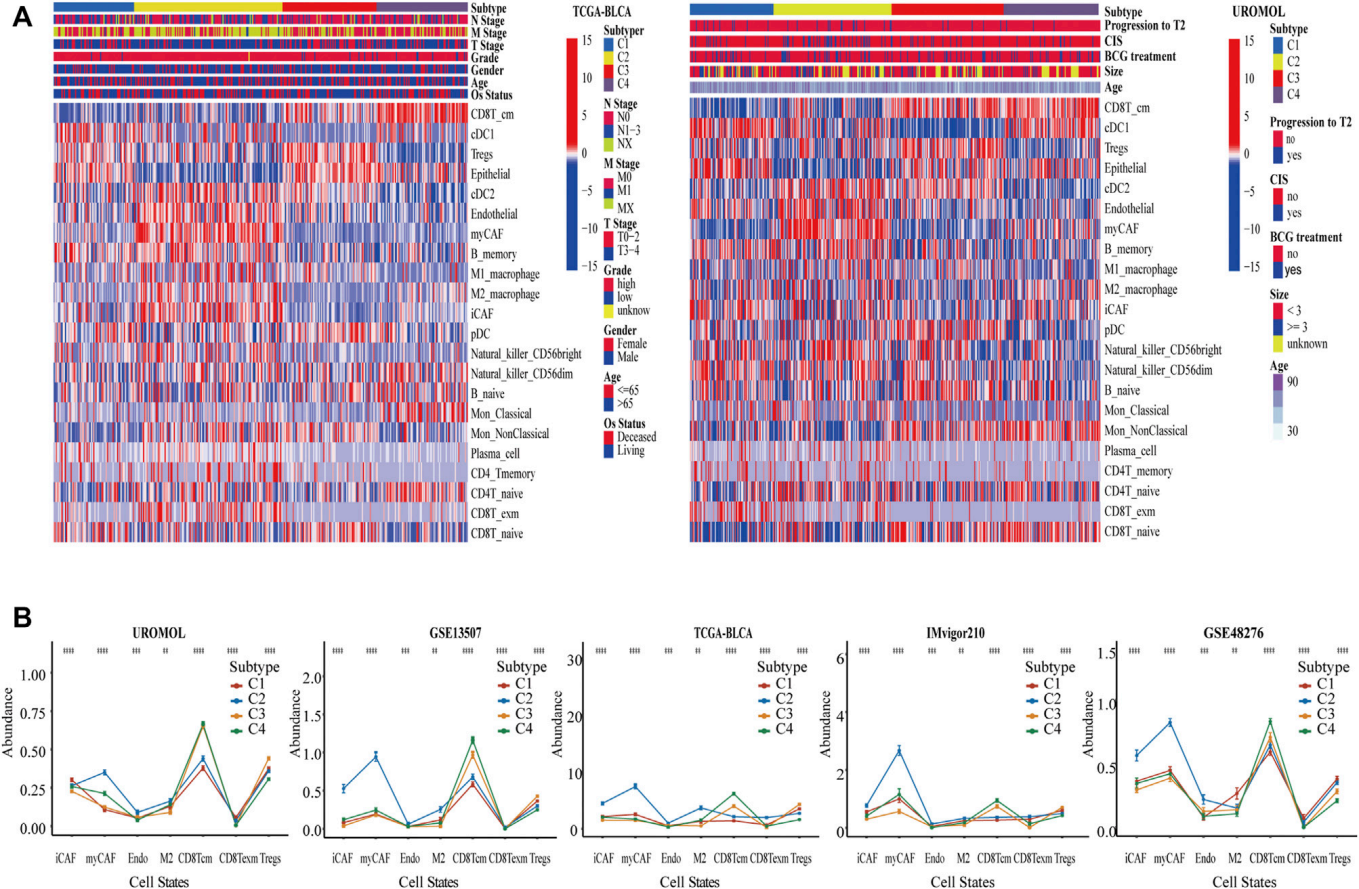
Identification of BC subtypes by clustering cell states. **(A)** Based on 22 cell states in BC, UROMOL, GSE13507, and GSE48276 cohorts were clustered into four subtypes, and was validated by TCGA-BLCA cohort et al. We only show two heatmaps of UROMOL and TCGA-BLCA cohorts. **(B)** The line graph demonstrates significant level of cell states among four subtypes in multiple cohorts. *p < .05, **p < .01, and ***p < .001. Abundance means the absolute proportion of cell states.

Based on the absolute proportion of 22 cell states, the CrossICC package was employed to cluster BC in UROMOL, GSE13507, and GSE48276 cohorts jointly. The cohorts from multiple platforms were intergrated using CrossICC with an iterative algorithm, and the collection of three training cohorts could be clustered into four subtypes, namely, C1, C2, C3, and C4. The results of clustering by cell states were consistent among three cohorts, which were validated in TCGA-BLCA, IMvigor210, and GSE31684 and presented with a heatmap in TCGA-BLCA, UROMOL cohorts (Figure 2A) and GSE13507 (Supplementary Figure S1); the identified results of BC subtypes among six cohorts are shown in Supplementary Table S3. The three heatmaps show that a pattern of four subtypes is consistent among the three cohorts. Moreover, a line graph was plotted to show significant differences of cell states in TCGA-BLCA, UROMOL, IMvigor210, GSE13507, and GSE48276 (Figure 2B). It can be found that C1 and C3 possessed the highest absolute proportion of Tregs, and the absolute proportion of endothelial cells in C2 and C3 was the highest, suggesting the tumor-related angiogenesis (Togashi et al., 2019). Besides this, the absolute proportion of iCAF, myCAF, M2_ macrophage, and CD8T_exm was the highest in C2, and that of CD8T_cm was the lowest in C2. The absolute proportion of CD8T_exm was the lowest in C3, and the absolute proportion of CD8T_cm was the highest in C4. These cell states not only showed a broadly consistent pattern in the training cohorts, but also showed similar pattern in the validation cohorts (Figure 2B). CAFs could accelerate tumor invasion by promoting tissue remodeling and EMT (Gaggioli et al., 2007; Astin et al., 2010) and release inflammatory factors to shape the immunosuppressive environment (Kaur et al., 2019). CD8T_exm highly express PD-1 and other immune inhibitors, which relate to poor prognosis (Ma et al., 2019), whereas CD8T_cm, which relates to good prognosis, could lead to protective immunity (Fraser et al., 2013; Olson et al., 2013). M2_ macrophage could facilitate abnormal angiogenesis by secreting pro-angiogenic factors as well as Tregs (Lee et al., 2020). Thus, four subtypes were characterized as described in Supplementary Table S4. C1 was defined as an immune suppressive subtype. C2 was defined as an EMT, angiogenic, immunosuppressive, and immune exhausted subtype. C3 was defined as an angiogenesis subtype, and C4 was defined as an immune effective subtype.

### Characterization of Clinical Features in Four Subtypes

**FIGURE 3:**
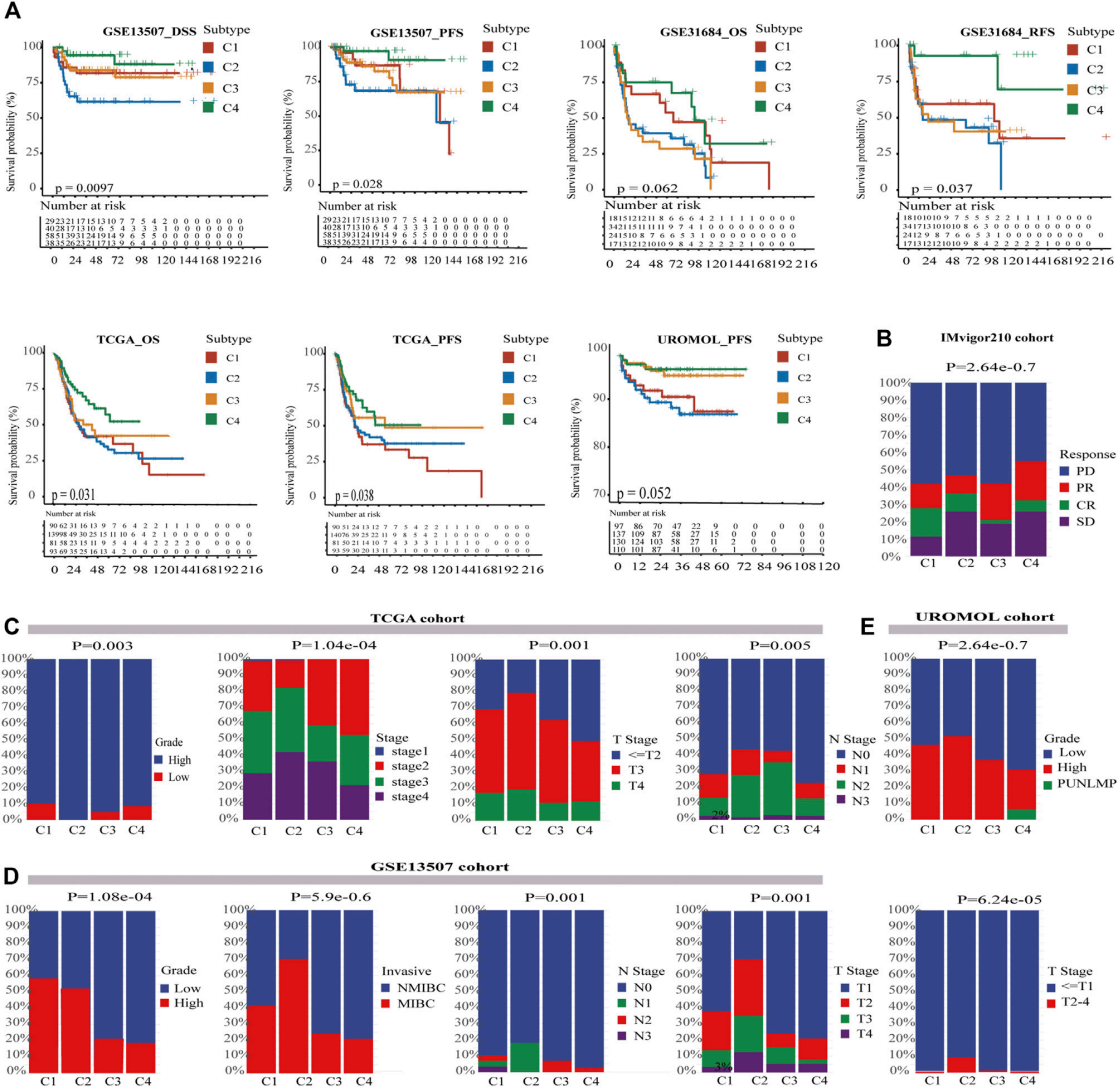
Characterization of clinical features in four subtypes. **(A)** Kaplan–Meier curves show four subtypes had survival differences in multiple cohorts, and C4 had the best prognosis while C2 has the worst prognosis. The log-rank test *p* values are shown. **(B)** The stratified bar chart shows the immune response rates for four subtypes; C1 and C4 both had the highest response rate (30%), and C2 had the lowest immune response rate (20%). C3 had the median immune response rate (23%) (chi-square test, *p* < .05). **(C)** In the TCGA-BLCA cohort, the clinical characteristics of C4 were more favorable for survival, whereas C2 was the opposite. C2 had most advanced tumors and most lymph node metastasis (chi-square test, *p* < .05). **(D)** In the GSE13507 cohort, the clinical characteristics of C4 were also more favorable for survival, whereas C2 was the opposite (chi-square test, *p* < .05). **(E)** In the UROMOL cohort, C4 also had better clinical characteristics than C2 (chi-square test, *p* < .05). PUNLMP means Papillary Urothelial Neoplasm of Low Malignant Potential.

The clinical features among four subtypes were compared to determine whether our cell-state subtyping method could distinguish clinical heterogeneity in BC. First, survival analysis was conducted on the training cohorts, which showed that there were significant differences in progression-free survival (PFS) of the UROMOL cohort and in PFS, and disease-specific survival (DSS) of the GSE13507 cohort (*p* < .05). Moreover, overall survival (OS) and relapse-free survival (RFS) in GSE31684 and OS and PFS in TCGA-BLCA also showed remarkable differences (*p* < .05). Strikingly, in all these cohorts, C4 had the best survival rate, whereas C2 had the worst survival rate (Figure 3A). Furthermore, we found that other clinical features were heterogeneous and associated with the survival in four subtypes. İn TCGA-BLCA and UROMOL cohorts, the proportion of high-grade tumors followed the same pattern: C2 > C1 > C3 > C4. Actually, high-grade tumors are often accompanied by poor prognosis. In the TCGA-BLCA cohort, T3 and above stages follow the pattern: C2 (79%) > C1 (69%) > C3 (62%) > C4 (50%) (Figures 3C,D). Besides this, stage, grade, and invasion of four subtypes showed the same pattern in GSE13507 as well as in TCGA-BLCA and UROMOL cohorts (Figure 3E). The IMvigor210 was a drug-resistance cohort after cisplatin treatment and was sequenced before administration of immunotherapy. We found that the percentage of ORR was higher in C1 (30%) and C4 (30%) and lower in C2 (20%) and C3 (23%) (*p* < .05) (Figure 3B). In conclusion, the above analysis suggests that C2 tend to be advanced tumors with the worst prognosis, whereas C4 is on the contrary. C1 and C4 are likely to respond better to immune therapy than C2 and C3.

### Characterization of TME, Inflammation, and Immunotherapy Heterogeneity by Signature in Four Subtypes

**FIGURE 4:**
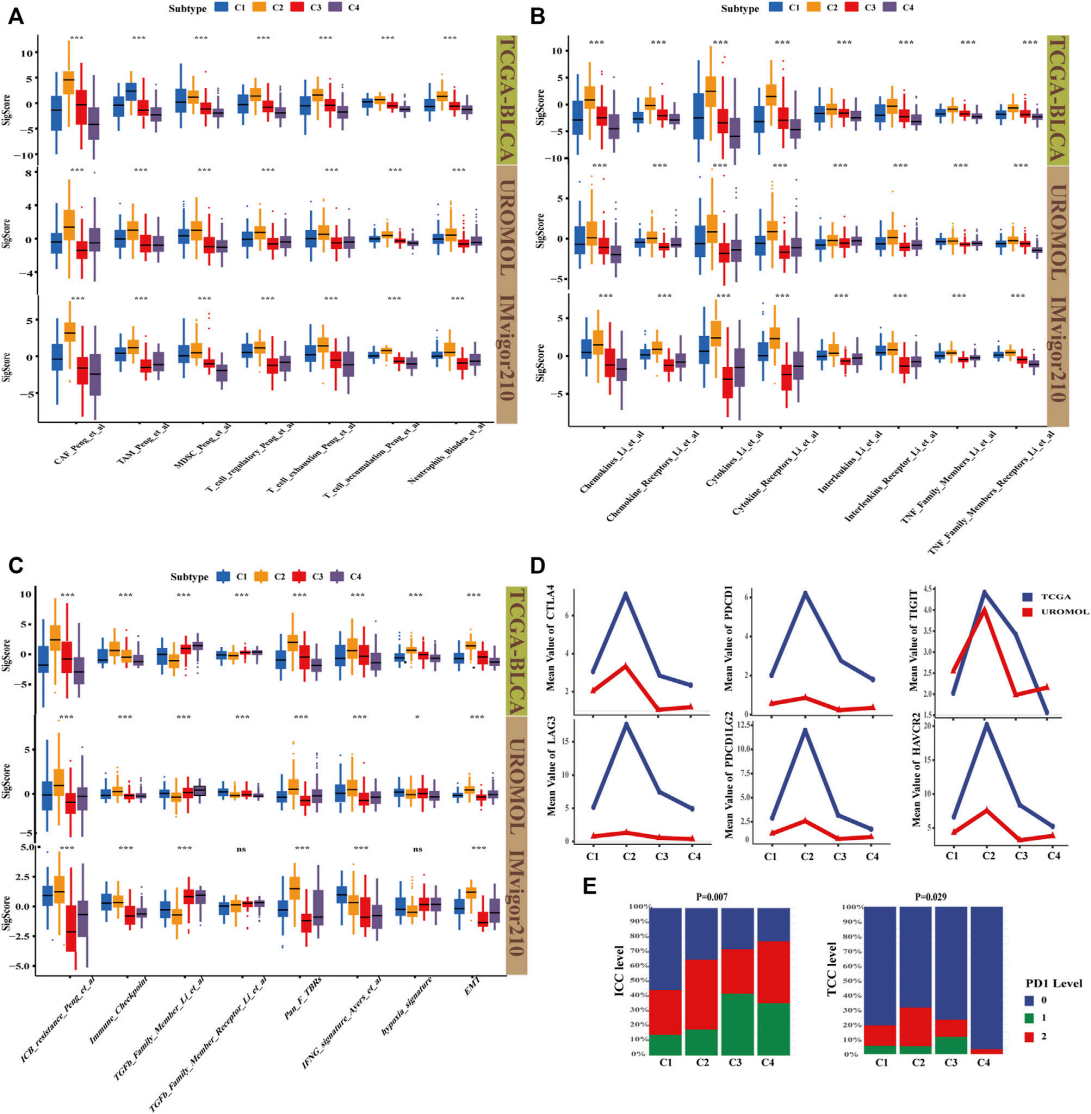
Characterization of TME, inflammation, and immunotherapy heterogeneity by signature in four subtypes. **(A)** Boxplot shows TME signature of four subtypes; C4 had the highest immune effective and the lowest immunosuppressive and immune exhausted signature, whereas C2 was the opposite. C1 had a relatively high immunosuppressive environment. Kruskal–Wallis (K–W) test was performed among four subtypes. *p < .05, **p < .01, and ***p < .001. **(B)** Boxplot shows inflammation signature correlated with chemokine, cytokine, interleukin, and TNF family. C2 had the highest tumor-associated inflammation, whereas C4 had the lowest. C1 and C3 had the median level. K–W test was performed among four subtypes. *p < .05, **p < .01, ***p < .001. **(C)** Boxplot showed immunotherapy signature, immune-resistant related signature and EMT signature were the highest in C4 but the lowest in C2. K–W test was performed among four subtypes. *p < .05, **p < .01, and ***p < .001. **(D)** The line graph shows the average gene expression of the six immune checkpoints, C2 had the highest level in all six immune checkpoints. **(E)** Hierarchical bar graph showed the PD1 level in immune cells (ICC) and tumor cells (TCC) in the IMvigor210 cohort (chi-square test, *p* < .05).

To investigate whether the four subtypes are heterogeneous in TME, immunotherapy, and inflammation, the signature score of three cohorts (TCGA-BLCA cohort (MIBC), UROMOL cohort (NMIBC), and IMvigor210 cohort (immunotherapy)) were calculated by using the IOBR package and displayed in Supplementary Table S5. Then, the characteristics of the four subtypes were compared, and significantly different TME signatures were displayed. İt was found that C2 has the highest level of T cell accumulation and also has the most immune exhaustion and immunosuppressive characteristics, such as CAFs, myeloid-derived suppressor cells (MDSC), tumor-associated macrophages (TAM), and Tregs, whereas C3 and C4 were almost the opposite. In terms of the level of immunosuppression, C1 is second only to C2, but C1 had low immune exhaustion. In addition, the neutrophil signature, which always means high levels of inflammation (Wu and Zhang, 2020), followed the same pattern in the three cohorts (Figure 4A). The results are largely consistent with cell states in four subtypes; this verified that our cell-state subtyping method could correctly distinguish the heterogeneity of TME. Next, the signature correlated with the inflammatory marker in these three cohorts was calculated to assess the level of inflammation; we were amazed to find that the level of inflammatory signature, including chemokines, cytokines, interleukins, and tumor necrosis factor family members follow the same pattern: C2 > C1 > C3 > C4 (Figure 4B). Inflammatory markers could mediate an immunosuppressive environment by inducing MDSC activation and proliferation (Parker et al., 2014; Bronte et al., 2016; Veglia et al., 2018), which explained poor prognosis in C2. Moreover, the highest chemokine-related signature in C2 means high chemokines, which could promote tumor metastasis (Romero-Moreno et al., 2019); this is consistent with that C2 was characterized as a high EMT subtype. Overexpression of inflammatory factors could increase neutrophil infiltration (Zhou et al., 2012), which may explain the highest percentage of neutrophil signature in C2 (Figure 4B). Furthermore, the signature related to heterogeneity of immunotherapy response among four subtypes was compared. Immune checkpoint blockade (ICB) resistance was considered to be able to predict immune resistance in patients (Jiang et al., 2018). It can be seen that C2 has the highest ICB resistance, whereas C3 and C4 had lower ICB resistance than C2 (Figure 4C). However, the immune response rate in C3 was almost as poor as that in C2 as above stated in the IMvigor210 cohort. A possible explanation was that C3 had the second highest proportion of endothelial, which means active angiogenesis. Tumor angiogenesis, which could result in immunosuppression and affect the response rate of immunotherapy, is described in a previous study (Rahma and Hodi, 2019). Then, we noticed that C2 with the lowest ORR unexpectedly had the highest immune checkpoint signature (Figure 4C). The mean value of six immune checkpoints, including PDCD1, PDCD1LG2, CTLA4, TIGIT, LAG3, and HAVCR2 followed the same pattern (Figure 4D). ORR was always positively correlated with the expression of immune checkpoints. To further explore this problem, the PD1 level of tumor cells and immune cells in the IMvigor210 cohort were analyzed, which exhibited that C2 had the third highest PD1 level in immune cells but had the highest PD1 level in tumor cells (Figure 4E). Actually, PD1 with high expression in tumor cells indeed exerted inhibitory effects on immunotherapy response (Zhao et al., 2018). Moreover, C2 had the highest IFNG_signature, which was said to be able to assess IFN-γ activity in fibroblasts and negatively correlated with the ORR of patients (Ayers et al., 2017). We also noticed that the total TGFβ signature was lowest in C2, whereas the Pan_F_TBRs signature, which is defined as a signature to quantitatively determine the level of TGFβ in pan-fibroblasts and positively correlated with immune tolerance, was the highest. The result indicates that TGFβ secreted by CAFs could predict immunotherapy response more accurately. C2 also had the highest hypoxia signature, but there were only significant differences of hypoxia signature in TCGA-BLCA cohort. In addition, the EMT signature related to metastasis was also the highest in C2. These results explain why C2 had the worst prognosis and immune response while C4 had the best prognosis, and why C3 had a poor immune response but the better prognosis. Of course, these results explain the possible reason for the high response rate of C1 and C4, which were considered to be the immunosuppressive-only subtypes.

### Characterization of Genomic Driver Mechanisms by Mutation Profiles in Four Subtypes

**FIGURE 5:**
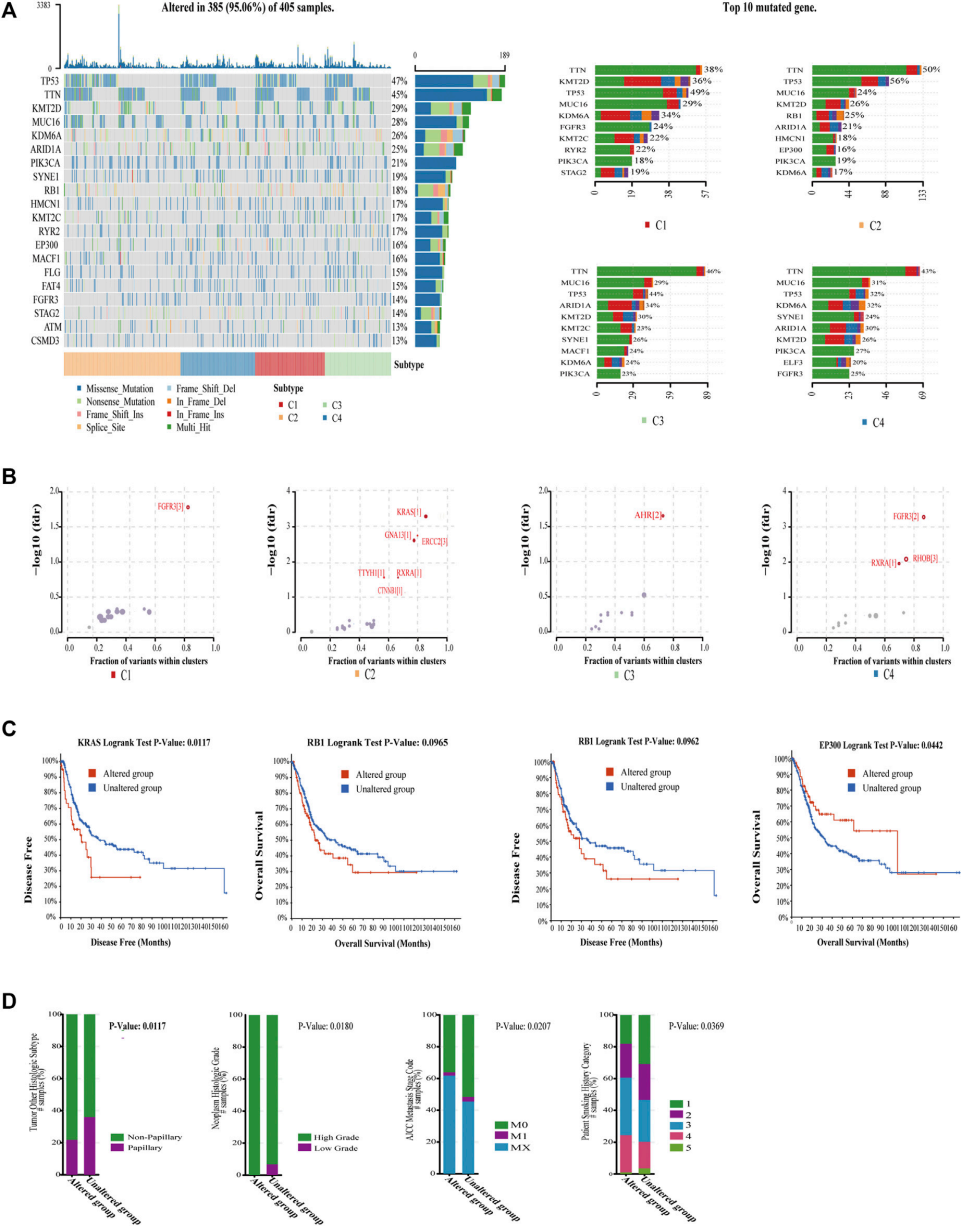
Characterization of genomic driver mechanisms by mutation profiles in four subtypes. **(A)** The vast majority of BC were mutated, and waterfall plots demonstrate significant differences in the mutation rates of the top 20 mutated genes in the overall cohort across the four subtypes as well as significant differences in the mutation rates of top 10 mutated genes in each subtype. **(B)** Bubble plot shows driver mutations of four subtypes, and C2 had most of the driver mutations, which mostly cause immunosuppression. **(C)** KRAS, RB1, and EP300 mutations, which are mostly mutated in C2 correlated with worse OS or DFS in TCGA-BLCA cohort. The log-rank test *p* values are shown. **(D)** RB1 mutation correlated with bad histologic subtype (chi-squared test, *p* = .0133), high grade (chi-squared test, *p* = .0180), high metastasis stage (chi-squared test, *p* = .0207), and more smoking (chi-squared test, *p* = .0369).

**FIGURE 6:**
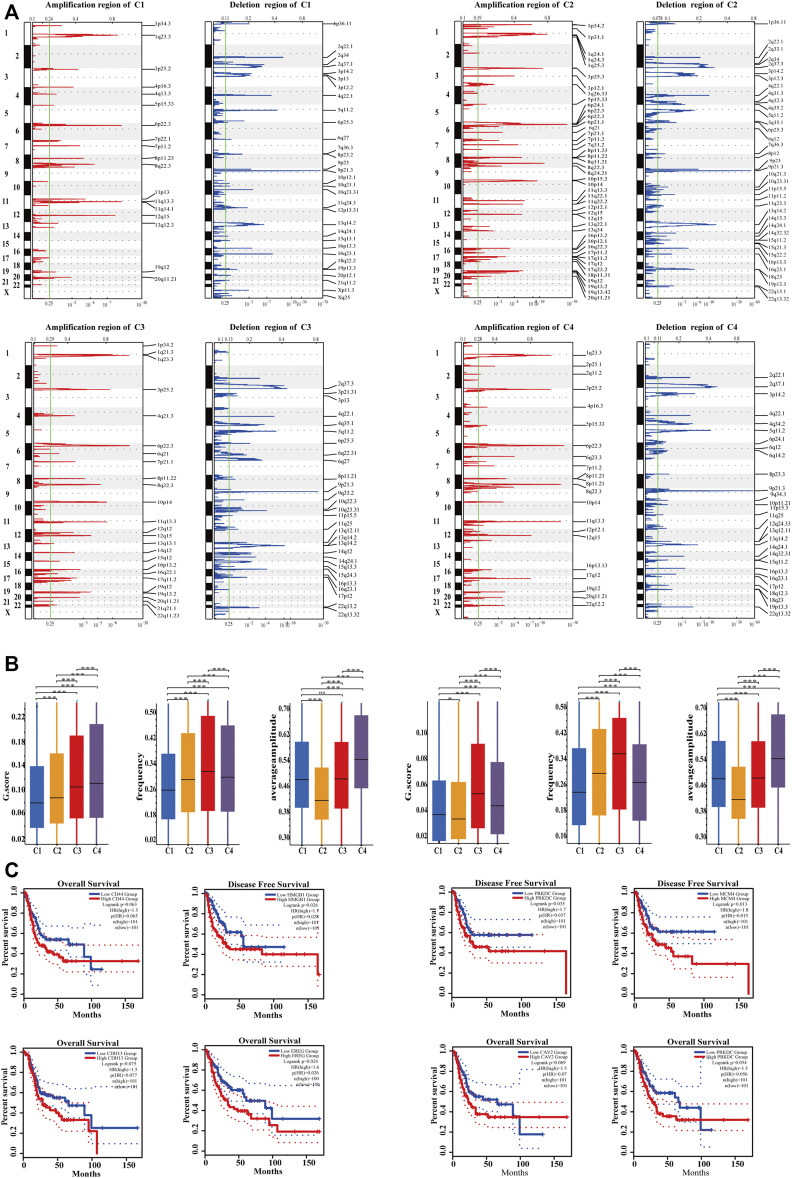
Characterization of genomic driver mechanisms by SCNAs in four subtypes. **(A)** The four subtypes had significantly different recurrent SCNA regions. Ordinates represent chromosomal regions. **(B)** The four subtypes had significantly different GISTIC score, mutation frequency, and average amplitude (Kruskal–Wallis test, *p* < .05). C4 had the highest SCNA level; C2 had the lowest SCNA level though it had the highest mutation frequency and the lowest mutation amplitude. **(C)** In the C2 subtype, the expression of highly amplified genes tended to correlate with poor prognosis. Cutoff value of high and low expression groups were 75% and 25%.

Somatic mutations are reported to play an extremely important role in the development of cancer, which may be related to immunophenotypes (Porta-Pardo and Godzik, 2016), and BC is one of the cancers with the highest mutational burdens. The mutation profiles among four subtypes were compared. As expected, the mutation landscape of four subtypes was significantly different (Figure 5A). We noticed that C4 had the lowest mutation rate, whereas C2 had the highest mutation rate in TP53. TP53 could support the differentiation of breast epithelial cells into a basal-like subtype in previous study (Munne et al., 2014). This is consistent with the result that C2 does have the most basal-squamous subtype in the following subtype comparison. Next, the oncodriveclust algorithm was utilized to analyze the driver mutations of the four subtypes (Tamborero et al., 2013); the detailed driver mutations are shown in Supplementary Table S6. We found that C2 had the most driver mutations (Figure 5B). KRAS driver mutations in C2 could shape the immunosuppressive environment by upregulating PD-L1 and inducing apoptosis in CD3-positive T cells (Chen et al., 2017). It is also associated with the downregulation of major histocompatibility class I (MHCI) molecules (Atkins et al., 2004; Koelzer et al., 2015), which means that antigen presentation is impaired, and not easy to be detected by the immune system in C2. As expected, C2 did have MHCI deletion in 6q12 (Figure 6A). C2 driver mutation CTNNB1 could also lead to immune escape (Korpal et al., 2019). In C3 driver mutations, AHR mutations could activate the pro-angiogenesis of macrophages in TME (Kubli et al., 2019), which explained the poor ORR despite the high level of PD1 in C3. It was also found that C4 had a higher mutation rate than C1 and C2 in PI3KCA. Accumulating evidence validates that tumors with PI3KCA mutations can benefit from immunotherapy (Nusrat et al., 2019; Qi et al., 2020). The driver mutation RhoB in C4 could promote tumor formation but also limit tumor invasion (Meyer et al., 2014), which was consistent with that C4 has the lowest EMT signature score. We also found the mutations, including KRAS, RB1, and EP300 in C2, were directly correlated with poor survival (Figure 5C). Moreover, the RB1 mutation was associated with adverse clinical features, such as histological subtype, high grade, high metastasis, and more smoking (Figure 5D). These mutations of C2 could be biomarkers for predicting poor prognosis and immunotherapy response in BC. These findings indicate genomic heterogeneity in four subtypes and could explain the genomic driver mechanisms leading to heterogeneity of prognosis and immunotherapy resistance.

### Characterization of Genomic Driver Mechanisms by SCNAs in Four Subtypes

Recently, Zhou et al. revealed the presence of SCNAs in immune cells, fibroblasts, and endothelial cells in human colorectal cancer (Zhou et al., 2020), indicating that SCNAs are associated with cell states in TME. The SCNAs of four subtypes in the TCGA-BLCA cohort were analyzed and compared, and the result showed that the four subtypes had different SCNAs (Figure 6A). The 13q12.3 amplification in C1 contained HMGB1, which could promote the development of MDSC and the metastasis of cancer cells (Gorgulho et al., 2019; Ren et al., 2021). FSCN1 amplification at 7p22.1, Gab2 amplification at 11q14.1, and CD44 amplification at 11p13 were also associated with promoting tumor invasion (Ke et al., 2007; Li et al., 2018; Rohani et al., 2019) and inducing tumor resistance to radiotherapy (Li et al., 2021), suggesting that patients with C1 had high immune suppression, active EMT, and may not be suitable for radiation therapy. The 19q13.42 amplification of C2 contained NLRP3, which could upregulate the expression of PD-L1 to form immunosuppression (Lu et al., 2021). The 11q22.2 amplification leads to high expression of MMP9 in C2, which can promote tumor cell migration and invasion (Li et al., 2020). Upregulation of VEGFA at 6p21.1 in C2 could increase the malignancy of tumor cells and was often accompanied by hypoxia, angiogenesis, and immunosuppressive TME, suggesting tolerance to immunotherapy (Wang et al., 2020). 9p21 deletion (CDKN2A/B) in C2 also conferred primary resistance to immune checkpoint therapy (Han et al., 2021). LAMC2 amplification at 1q25.3 could result in gemcitabine resistance via EMT (Okada et al., 2021). It can be demonstrated that C2 had high EMT, immunosuppressive and angiogenesis from genomic SCNAs, and PD-1/PD-L1 blockade therapy should be combined with anti-angiogenesis therapy in C2. Moreover, four subtypes had significantly different GISTIC score, mutation frequency, and average mutation amplitude (Kruskal–Wallis test, *p* < .05). C4 had the highest SCNAs level, and C2 had the lowest SCNAs level; although the mutation frequency of C2 was the highest, its mutation amplitude was the lowest (Figure 6B). The result was consistent with that tumors containing activated KRAS mutation showed less SCNAs in C2 but was inconsistent with that tumors with less SCNAs showed poor survival (Davoli et al., 2017). As described in a previous study, SCNAs often play a regulatory role in gene expression independently (Sun et al., 2018). It was found that gene expression of highly amplified genes in C2 was correlated with poor prognosis (Figure 6C).

### Characterization of Associations Between the Cell-State subtyping and Other Subtyping Methods in BC

**FIGURE 7:**
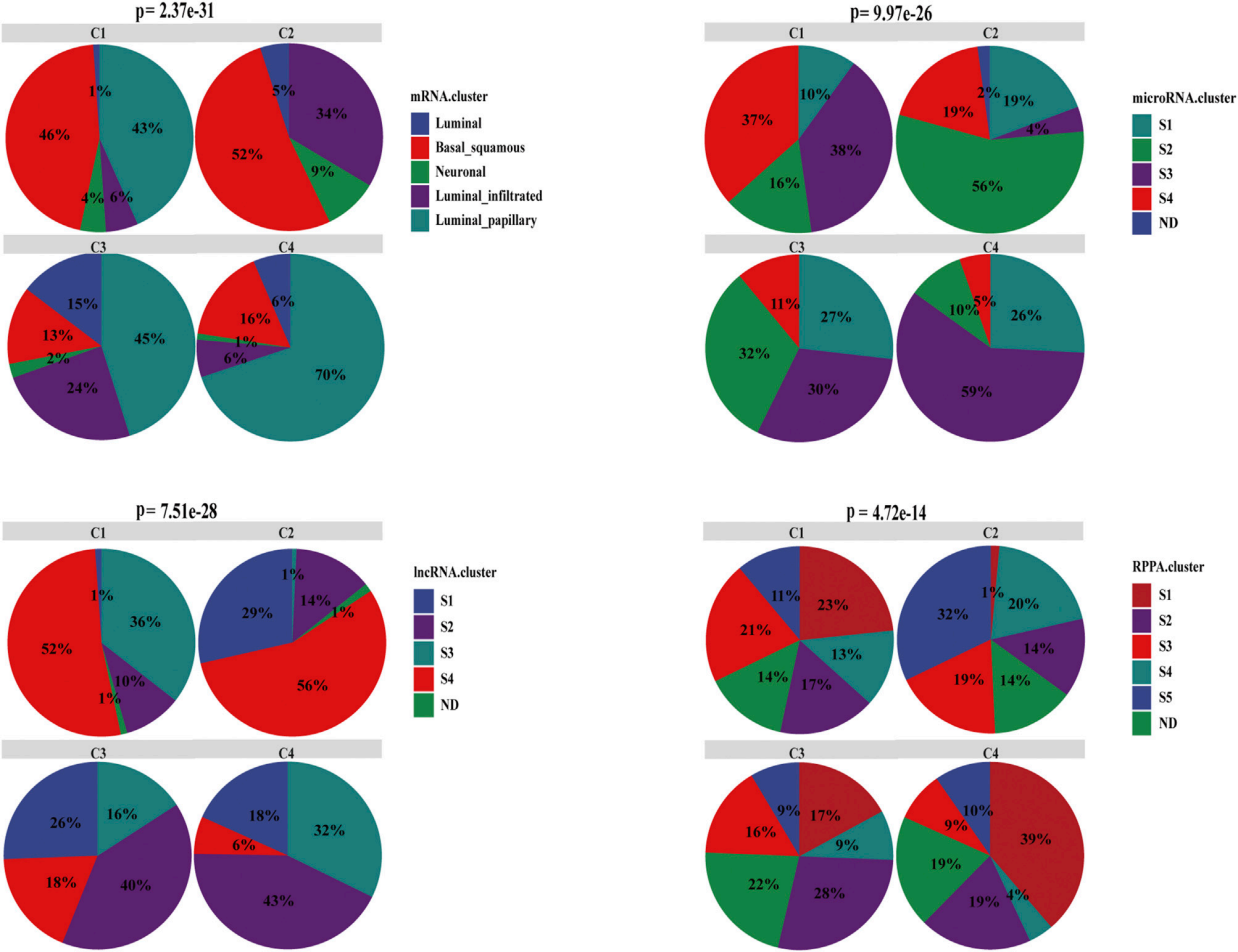
Characterization of associations between the cell-state subtyping and other subtyping methods in BC. Compared with mRNA subtype, basal_squamous was enriched with C1 and C2, and luminal_papillary was enriched with C3 and C4. Compared with miRNA and lncRNA subtypes, C2 had the least miRNA S3 subtype and lncRNA S3 subtype, C1 was the second least, whereas S3 subtype had the best prognosis and low EMT. Conversely C1 and C2 had the most S4 subtype, and the S4 subtype correlated with relatively high EMT and always accompanied the worst prognosis. In comparison with RPPA subtype, C4 was dominated by S1 subtype, which indicated good prognosis and had the least C2, and conversely C4 had the least S4 subtype, which was correlated with the worst prognosis. ND meant that subtype was not determined or unknown.

Our cell-state subtyping method was compared with previous subtyping methods, including mRNA, miRNA, lncRNA, and RPPA subtyping methods in TCGA-BLCA (Robertson et al., 2017), which showed that C2 was dominated by basal subtype (52%), whereas C1 was dominated by both basal (46%) and papillary subtype (chi-square test, *p* < .05). Patients with basal subtype always had basal and squamous differentiation markers, and prognosis was poor (Robertson et al., 2017), C2 had the worst prognosis indeed. In addition, the mutation rate of TP53 in the basal subtype was the highest (Robertson et al., 2017), which was consistent with the result that the mutation rate of TP53 in C2 was the highest among the four subtypes. C4 had the most luminal papillary subtype (70%), C1 and C3 have about 40% luminal papillary subtype, whereas no luminal papillary subtype was found in C2. Luminal papillary subtype may develop from NMIBC with lower stage and higher purity, which is related to good prognosis (Robertson et al., 2017). Moreover, the miRNA and lncRNA S3 of C2 was the least, followed by C1 (chi-square test, *p* < .001). Both miRNA and lncRNA S3 subtypes were positively correlated with the good prognosis and low EMT. In contrast, C1 and C2 were mainly included in S4 subtype, whereas the S4 subtype was associated with relatively high EMT with the worst prognosis, which was consistent with the characterization of C1 and C2. Compared with the RPPA subtyping method, C4 were enriched with the most S1 subtype and the least S4 subtypes (chi-square test, *p* < .05), which suggests good prognosis, whereas C2 was exactly the opposite (Figure 7). These results were also consistent with the features of four subtypes as described above and suggest that our cell-state subtyping method could be a supplement to previous subtyping methods.

## Discussion

Recently immune checkpoint blockade drugs such as atezolizumab and nivolumab are increasingly being used for immunotherapy of BC. However, due to the heterogeneity of TME, immune checkpoint blockade therapy is only effective in a few patients (20%–30%). To further understand the heterogeneity of BC TME and the relationship between immunotherapy resistance and TME and find suitable patients for immune checkpoint blockade, a subtyping method based on cell states in BC is proposed for the first time in this study.

In the present study, BC was classified into four subtypes based on 22 cell states in multiple cohorts. C1 was characterized by high Tregs and M2_macrophage, indicating that C1 had a suppressive environment. C1 responded to immune checkpoint blockade well and had a relatively better prognosis than C2. C2 was characterized by high CAFs, endothelial, M2_macrophage, Tregs, CD8T_exm, and low CD8T_cm, which suggests that C2 was enriched in EMT, angiogenesis, immune suppressive, and immune exhausted. C2 had the lowest immune response rate and the worst prognosis. C3 was characterized by the highest endothelial cells and lower CD8T_exm, suggesting that C3 was an angiogenesis-enriched subtype. The prognosis of C3 was better than that of C2, but the immune response rate was as low as that of C2. C4 was characterized by high CD8T_cm and moderate immunosuppression, which means that C4 was an immune effective subtype with the best prognosis and highest immune response rate.

In general, patients with high PD1 expression had a strong response to immune checkpoint blockade therapy, whereas C2 with high expression in all six immune checkpoints unexpectedly had the lowest immune response rate. A prior study reported that tumor cells can express PD-1 and bind to their own PD-L1, contributing to failure of immune checkpoint blockade therapy (Zhao et al., 2018). We found that C2 immune cells secreted relatively high levels of PD-1, and C2 tumor cells expressed the highest level of PD-1, which further supports this conclusion. It can be concluded that the PD-1 level of both immune and tumor cells can affect the effect of immune checkpoint blockade. The IMvigor210 cohort concluded that only the PD-1 level of immune cells affects the effect of immune checkpoint blockade, which requires further studies to resolve this issue. İt is also interesting to note that C2 displayed low levels of overall TGFβ but high levels of Pan_F_TBRs, whereas C4 was the opposite. Although the level of overall TGFβ in C4 was high, CAFs did not express TGFβ highly in C4, whereas it expressed TGFβ highly in C2. This result suggests CAF-specific TGFβ could predict immunotherapy resistance more accurately and the heterogeneity of biomarkers in tissue distribution. Therefore, the indication of immunotherapy should consider not only the overall expression of immune checkpoints, but also the level of immune checkpoints in specific cell states. By comparing C1 and C3, we believe that the main reason for the poor response in C3 is abundant abnormal angiogenesis, suggesting that the level of angiogenesis needs to be checked before using immune checkpoint inhibitors. Transcriptomic and genomic alterations in four subtypes were consistent with the features of cell states, which indicates that the cell-state subtyping method can distinguish transcriptomic and genomic heterogeneity. In addition, Bacillus Calmette-Guérin (BCG) have no impact on our cell-state subtyping in the UROMOL discovery cohort as presented (Figure 2A; Supplementary Table S7). None of the patients received any other treatment besides BCG, and the patients only received BCG prior to collection of the analyzed samples in the UROMOL cohort. This is consistent with the finding of the UROMOL study that no significant difference in the BCG treatment and response to BCG were observed between UROMOL classes (Supplementary Table S8, S9). The possible reasons to explain this are that patients of the UROMOL cohort were included from several hospitals and had different clinical risk among the hospitals. More cohorts and experiments related with BCG are needed to be reasonably designed and studied to explore this problem.

Previous studies identify many subtypes of BC, such as subtypes identified by mRNA, miRNA, lncRNA, and RPPA in TCGA-BLCA. Compared with these subtyping methods, our cell-state subtyping method offers some advantages. First, previous TME subtyping methods were mostly based on a specific marker panel (Bagaev et al., 2021). These markers are almost nontissue-specific molecules with a limited number, and the overall level of these markers was usually used in subtyping. Our cell-state subtyping method combined with single cell RNA-seq, RNA microarray data and bulk RNA-seq to obtain cell states that reflect the integration of thousands of markers. Evidently, our method is more comprehensive than previous studies. Besides this, our method also has higher tissue specificity for the usage of single cell RNA-seq in BC. Furthermore, previous studies mostly focus on immune cells, and we focus not only on immune cells, but also on nonimmune cells, including endothelials and fibroblasts. Moreover, our subtyping approach can distinguish the heterogeneity of many clinical features, including ORR, and which suggests that our subtypes can well guide the use of immunotherapy. Finally, previous BC subtyping methods mostly include only one cohort. For example, TCGA used multi-omics data but from a single cohort. However, about seven cohorts were used in our subtyping method, including NMIBC and MIBC. In all of these cohorts, our subtypes can distinguish their molecular and clinical characteristics well, indicating the robustness of our cell-state subtyping method.

## Conclusion

In summary, from a novel perspective of cell states, single cell RNA-seq combined with RNA microarray and bulk RNA-seq was conducted to classify BC into four subtypes, which could distinguish the heterogeneity of transcriptome, genome, clinical characteristics, and TME in BC. Our subtyping method explored the relationship between TME and immunotherapy resistance and indicated that cell states could be biomarkers to predict prognosis and immunotherapy response, which is helpful to develop flow cytometry for aiding diagnosis and further guide treatment using postoperative cancer tissues. Our work is expected to attract more attention to study cancer from comprehensive cell states, not just molecules, because TME is composed not only of molecules, but also of cells.

## Data Availability

Publicly available datasets were analyzed in this study. The names of the repository/repositories and accession number(s) can be found in the article/Supplementary Material.

## Footnotes

1
https://www.ncbi.nlm.nih.gov/sra/.

2
https://portal.gdc.cancer.gov/.

3
https://www.ncbi.nlm.nih.gov/geo/.

4
https://www.ebi.ac.uk/arrayexpress/.

5
http://www.cbioportal.org/.

6
http://gepia2.cancer-pku.cn/.
