# Supplementary figures and images for: Multi-Omics Characterization of Tumor Microenvironment Heterogeneity and Immunotherapy Resistance Through Cell States–Based Subtyping in Bladder Cancer
Source: Front Cell Dev Biol. 2022 Feb 9;9:809588. doi: 10.3389/fcell.2021.809588 (PMC8864284; doi:10.3389/fcell.2021.809588)

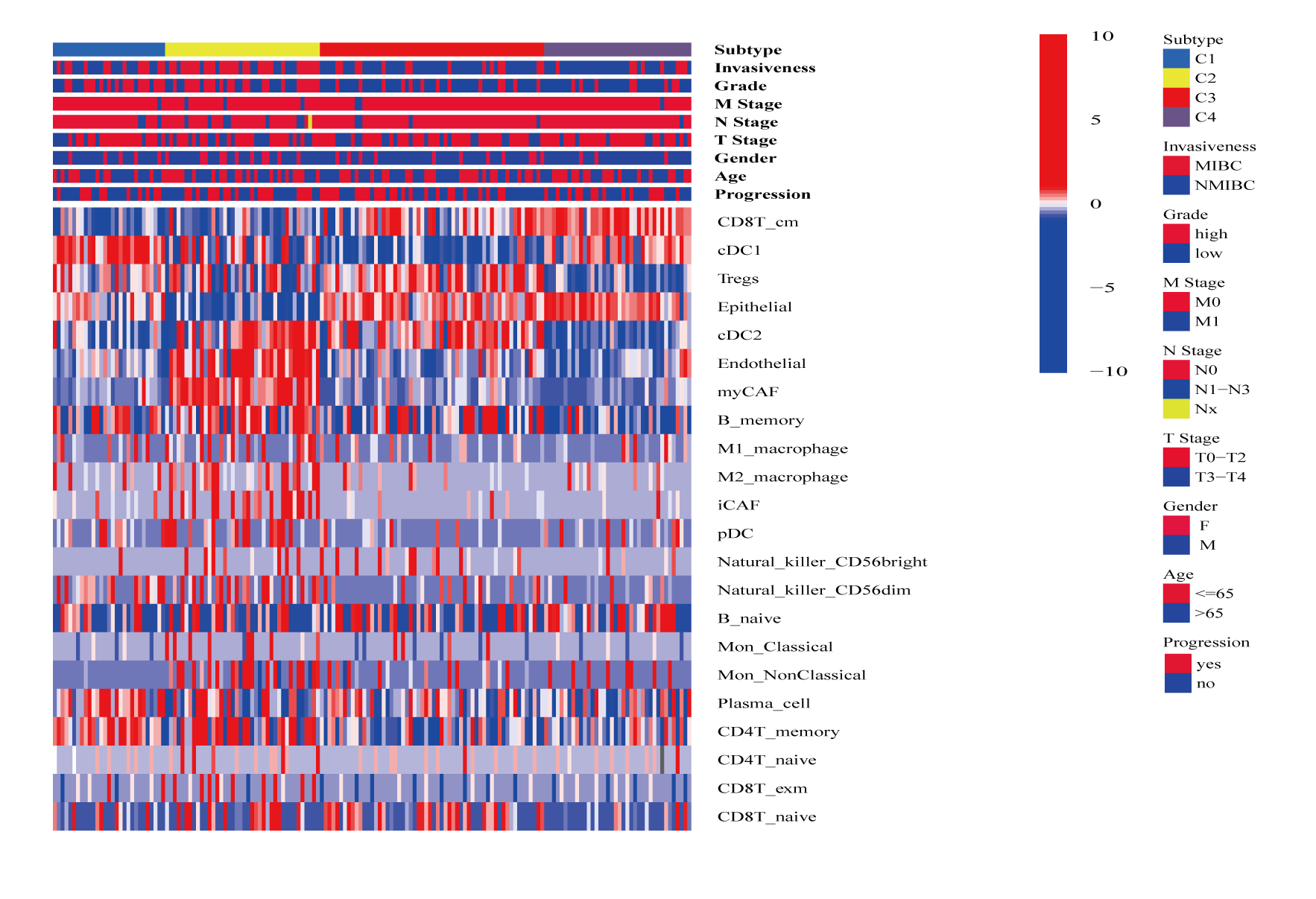

Supplement: Supplementary file 4 [file Image1.tif]
